# Capillary Electrophoresis Single-Strand Conformational Polymorphisms as a Method to Differentiate Algal Species

**DOI:** 10.1155/2015/272964

**Published:** 2015-05-26

**Authors:** Alice Jernigan, Christa Hestekin

**Affiliations:** Department of Chemical Engineering, University of Arkansas, Fayetteville, AR 72701, USA

## Abstract

Capillary electrophoresis single-strand conformational polymorphism (CE-SSCP) was explored as a fast and inexpensive method to differentiate both prokaryotic (blue-green) and eukaryotic (green and brown) algae. A selection of two blue-green algae (*Nostoc muscorum* and *Anabaena inaequalis*), five green algae (*Chlorella vulgaris, Oedogonium foveolatum, Mougeotia* sp., *Scenedesmus quadricauda*, and *Ulothrix fimbriata*), and one brown algae (*Ectocarpus* sp.) were examined and CE-SSCP electropherogram “fingerprints” were compared to each other for two variable regions of either the 16S or 18S rDNA gene. The electropherogram patterns were remarkably stable and consistent for each particular species. The patterns were unique to each species, although some common features were observed between the different types of algae. CE-SSCP could be a useful method for monitoring changes in an algae species over time as potential shifts in species occurred.

## 1. Introduction

The two most frequently used traditional methods for taxonomic identification of algae beyond the genus level are by microscopic examination and genotyping [1–5]. Microscopic examination methods require skill and experience as they involve a thorough knowledge of the thousands of possible variations in the shape and morphology of the algae as well as familiarity with the algae ecology and reproduction [6]. To go beyond the genus level to species or substrains of a species can require a scanning electron microscope (SEM) or other more complicated tests and techniques such as in the case of diatoms [7]. Even at this level, there may still be some ambiguity and discussion among the experts in the field of phycology.

Genotyping, in which the DNA is extracted and sequenced, is another method. While giving excellent results, it requires skill and takes time and if hired out to a laboratory that specializes in sequencing, it can be costly. Genotyping requires primers that can isolate and amplify a portion of the genome of the species of interest, typically the 16S rDNA gene [8–10] for prokaryotic algae (cyanobacteria or blue-green algae) and the 18S rDNA gene [11] for eukaryotic algae. These genes are both highly conserved among algae but have regions of variability that can be used for species identification [2, 11–13]. The sequence of these regions can then be compared to known sequences and the most likely species matching sequence can be determined.

While sequencing is the gold standard method for evaluation of sequence variants, alternative methods with more rapid analysis times and lower expense are also being evaluated for their potential. One such method is capillary electrophoresis single-strand conformational polymorphisms (CE-SSCP). SSCP entails heat denaturing the double stranded DNA until the hydrogen bonds holding the double helix backbone together disassociate and the DNA becomes two single strands in a dilute solution. These single strands of DNA are snap-cooled on ice causing the single strand to fold back on itself in ways that are dictated by its unique nucleotide sequence into something called a conformer. Under conditions of low temperature and nondenaturing polymer the single-stranded DNA conformers will migrate according to their shape and will have a distinct mobility. SSCP performed on a capillary electrophoresis instrument gives the advantages of automated loading of samples and the use of laser-induced fluorescence detection, which allows for sensitive detection at low concentrations and repeatable results.

CE-SSCP has been used to successfully differentiate between mutated and nonmutated genes [9, 16–18] and also to identify and differentiate bacterial species from such diverse samples such as blood, cheese, and soil [14, 19–21]. In addition, Herrera-Supúlveda used CE-SSCP to monitor activities of harmful algal blooms of the Baja California Sur Coastal waters and noted significant advantages of this method over having to keep on hand an expert taxonomist [22]. In this research capillary electrophoresis single-strand conformational polymorphism (CE-SSCP) was explored to identify different algal species.

## 2. Materials and Methods

### 2.1. Pure Cultures

Six pure species level cultures and two genus level cultures were obtained from Carolina Biological Supply Company (Burlington, NC, USA). The eight different cultures represented three different types of algae including green algae, brown algae, and cyanobacteria.  Table 1 gives a list of the various types of algal species used for the fingerprint database.

The live cultures were used for DNA extraction within a week of arrival. DNA was extracted using either the PowerPlant Pro DNA Isolation Kit from Mo Bio Laboratories (Carlsbad, CA, USA) or the DNeasy Plant Mini Kit from Qiagen (Germantown, MD, USA). The PowerPlant and the DNeasy Plant Mini Kit protocols were followed to obtain clean DNA samples for PCR.

### 2.2. Reagents and Primers for PCR Amplifications

The PCR reagents 5x GoTaq Flexi Buffer, 25 mM MgCl_2_, 10 mM dNTP, and Taq polymerase were obtained from Promega (Madison, WI, USA) and all of the PCR primers used were obtained from Invitrogen (Carlsbad, CA, USA). Forward (sense) primers were fluorescently labeled with FAM (absorption 494 nm, emission 522 nm) and reverse (antisense) primers were fluorescently labeled with HEX (absorption 535 nm, emission 553 nm) to enhance the ease of identification between the forward and reverse single-strand peaks in the electropherograms. Once the primers were obtained, they were diluted to a stock concentration of 100 *μ*M and this solution was used to make a working solution of 20 *μ*M for the PCR reactions. After preparing the PCR mixture, it was placed in an Apollo ATC401 thermocycler (Ramsey, MN, USA). A fragment of either the 16S or 18S gene variable regions was amplified using the primer pairs shown in Table 2.

The appropriate fluorescently labeled primer pairs were used in a PCR reaction mixture (20 *μ*L) consisting of 14 ng of DNA, 1X GoTaq Flexi Buffer, 0.2 mM dNTP, 20 *μ*M primer (forward and reverse), 2.0 mM MgCl_2_, and 2.5 U of GoTaq polymerase (Promega Corp., Madison, WI, USA). After gently pipetting to mix and spinning the mixture for ~15 seconds to remove bubbles, the PCR mixture was subjected to the following amplification cycles on an Apollo ATC401 thermocycler: 95°C for 2–5 minutes, 30–35 cycles each of 95°C for 30–45 seconds, 5°C below the melting temperature of the primer with the lowest temperature for 30–45 seconds, and 72°C for 45–60 seconds, followed by one cycle of final extension step at 72°C for 5–20 minutes, with temperature, time, and cycle ranges based on an optimization of the program and the melting temperatures of the different primer pairs.

### 2.3. PCR Amplified DNA Samples Purification, Quantification, and Storage

Amplified DNA fragments were purified using the QIAquick-Spin PCR Purification Kit (Qiagen Inc., Valencia, CA, USA) following the manufacturer's instructions. Quantification of purified DNA was determined with a Nanodrop 1000 spectrophotometer (Thermo Scientific, Wilmington, DE, USA) based on the absorbance of DNA at 260 nm. The purified samples were used as a stock solution for preparing SSCP samples and were stored at −20°C.

### 2.4. CE-SSCP Sample Preparation

The fluorescently labeled purified DNA stock solutions were diluted to 1 ng/*μ*L with 10 mM Tris-HCL (pH 8.0) to obtain a 15 nM solution for SSCP. Ten *μ*L of the diluted, purified DNA was loaded per well into 4 wells of a 96-well plate followed by a denaturing step at 95°C for 3 minutes on the ATC 401 thermocycler. The samples were immediately snap-cooled on ice for 3 minutes before loading onto the ABI 3130 Genetic Analyzer (Applied Biosystems, Foster City, CA, USA) which has a four-capillary array. The capillaries were 36 cm in length with an inner diameter of 50 *μ*m. The capillaries were loaded with the 3.5% PDMA polymer for 2 to 3 min and preelectrophoresis was performed for 3 min at 417 V/cm. The DNA samples were injected at 333 V/cm for 15 seconds and separated at 417 V/cm (15 kV) with an associated current of 37.5 mA at 25°C. The ABI 3130 utilizes LIF detection with a 488 nm Showa Laser (25 mW, 7.5 amps) and has an electrokinetic injection method.

### 2.5. Preparation of Polymer

Polydimethylacrylamide (PDMA) polymer was synthesized using the method of Albarghouthi et al. except with a decrease in the nitrogen bubbling time (from 16 hours to 4 hours) [23]. After completion of the reaction, the polymer was purified by dialysis in deionized water using Spectra-Por cellulose ester dialysis membranes (Spectrum, Gardena, CA, USA), having a molecular mass cutoff of 1000 Da. The deionized water was changed 10 times in 5 to 10 days. After dialysis was complete, the polymer was frozen at −80°C for 48 hours and then recovered by lyophilization (Labconco, Kansas City, KS, USA). Dry polymers were dissolved in the running buffer which was 1x TBE buffer + 10% glycerol (89 mM Tris-base, 89 mM boric acid, 2 mM EDTA, and 10% glycerol) to obtain polymer concentrations of 3.5% (w/v). The diluted polymer was stored at room temperature.

## 3. Results

### 3.1. Pure Culture “Fingerprints”

**Figure 1:**
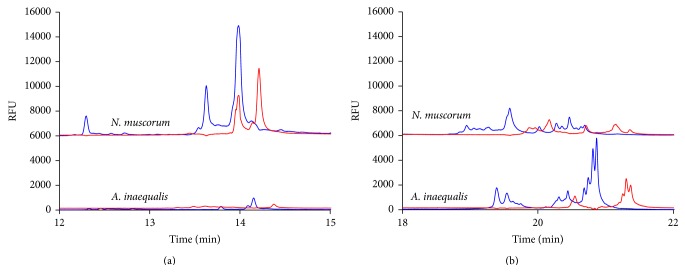
Detection of the (a) early and (b) later migrating peaks formed from the 16S rDNA gene variable region 2 as detected by CE-SSCP for the cyanobacteria (prokaryotic)* N. muscorum* and* A. inaequalis* samples.

For each type of algae (prokaryotic or eukaryotic), two different variable regions were analyzed by CE-SSCP. For the prokaryotic algae (cyanobacteria), variable regions 2 and 3 of the 16S rDNA gene were analyzed.  Figure 1 shows the electropherograms of the SSCP products of variable region 2 of the 16S rDNA gene of two prokaryotic algae species* Anabaena inaequalis* (*A. inaequalis*) and* Nostoc muscorum* (*N. muscorum*). Since multiple peaks were detected over a significant range of migrations times, the electropherograms were split into two different graphs. It should be noted while migration times can vary slightly from run to run due to the injection of fresh polymer, for a set of runs, the RSD value was 0.39%.

The peak patterns in Figure 1(a) show a marked difference between the two species. There are three very strong forward (blue) and two reverse (red) peaks between the 12- to 15-minute migration time for* N. muscorum*, while* A. inaequalis* had only one very small forward and reverse peak during this same migration time.  Figure 1(b) is a continuation of this graph and shows the later migrating single-stranded DNA conformers. Again there were significant differences between the peak patterns.* N. muscorum* generated ten small forward peaks and five small reverse peaks, while* A. inaequalis* had eight larger forward peaks and three reverse peaks. Both* N. muscorum* and* A. inaequalis* demonstrated multiple peaks which were often in clusters. In an ideal CE-SSCP electropherogram, there would be a single peak for each species. Multiple peaks that are very close together, as seen in Figure 1(b) especially, could indicate that the “pure” culture sample was actually multiple subspecies with slight variations in the sequence. Alternative, multiple peaks have been previously detected for pure DNA samples where the sequences allowed for multiple highly favorable conformations to be formed during the snap-cooling process [24]. The same amount of primer was used in each reaction, so the amplification efficiencies of the different algae DNA templates may have resulted in different electropherogram peak intensities. There is also the possibility of multiple and/or different copies of the same 16S rDNA gene in some species. This could explain the differences in intensities and/or the presence of multiple peaks in the algae samples. The presence of multiple peaks is not a result of the simultaneous amplification using two fluorescently labeled primers. The electropherograms produced from amplifications with one labelled and one nonlabelled primer were identical to those produced from each strand in the dual labeled samples.

**Figure 2:**
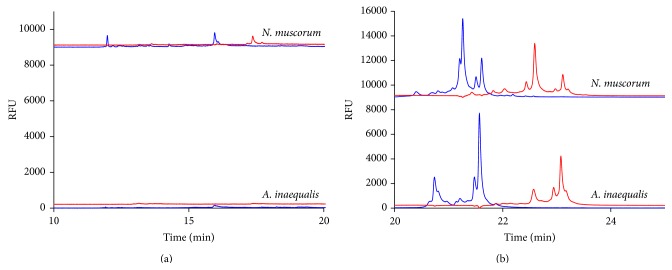
Detection of the (a) early and (b) later migrating peaks formed from the 16S rDNA gene variable region 3 as detected by CE-SSCP for the cyanobacteria (prokaryotic)* N. muscorum* and* A. inaequalis* samples. Forward or sense strands are represented in blue while reverse or antisense strands are represented in red.

The CE-SSCP electropherograms for the second 16S rDNA gene variable region examined (variable region 3) are shown in Figure 2.  Figure 2(a) shows the early migrating peaks (between 10 to 20 min); there are two very small forward peaks and one reverse peak for* N. muscorum* while* A. inaequalis* has no discernable peaks. In Figure 2(b),* N. muscorum* has four larger forward peaks and five reverse peaks. The reverse strands of both* N. muscorum* and* A. inaequalis* are at approximately the same migration time, but* N. muscorum* has a small peak at the 22-minute mark.* A. inaequalis* has four forward peaks in the 20- to 24-minute migration time period.

These two peaks at 21.5 minutes look very similar to the same peaks for* N. muscorum*. However, the first set of peaks for* A. inaequalis* at the 20.8-minute time has a difference in the migration time giving a slightly different pattern between the two species. The electropherograms or “fingerprint” patterns produced by these two cyanobacteria are extremely stable and the differences between them are repeated in all of the duplicated CE-SSCP runs (*n* ≥ 4). Therefore, these “fingerprint” differences clearly demonstrate how the electropherogram pattern could be used to differentiate between these two species. However, while the distinct pattern of peaks was very repeatable, the multiple peaks can create difficulties involved in interpreting these patterns as they can mask separate peaks and make the pattern difficult to read. Two different regions of each gene were examined to ensure verification of separate patterns for each species. For these two prokaryotic algae, the 16S rDNA gene variable region 2 provides the clearest differences in CE-SSCP patterns between the two species, although both variable regions could be used to differentiate them.

**Figure 3:**
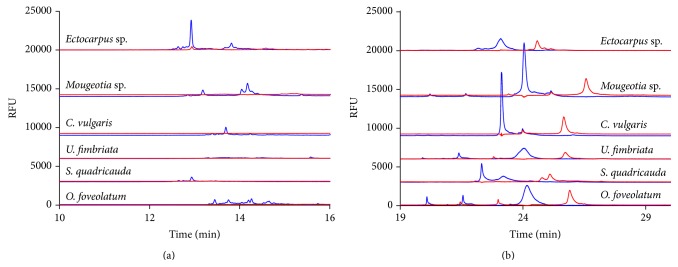
Detection of the (a) early and (b) later migrating peaks formed from the 18S rDNA variable region 7 as detected by CE-SSCP for green and brown algae. Forward or sense strands are represented in blue while reverse or antisense strands are represented in red.

After comparing two species of cyanobacteria using two variable regions of the 16S rDNA gene, we looked at several species of eukaryotic algae. The eukaryotic algae are of a higher cell complexity and have an 18S rDNA gene in their nucleus. Therefore the DNA sample is from the 18S rDNA gene variable regions 7 and 9, respectively.  Figure 3 provides a comparison of six species shown in the same graph to highlight the differences between both similar and dissimilar species of algae and how CE-SSCP can be used to distinguish between them. Four of these eukaryotic species belong to the phylum Chlorophyta and two belong to the class Chlorophyceae which is one of the classes of green algae [6], while one is in the phylum Charophyta (a conjugating algae), and one is in the phylum Ochrophyta (a brown algae) [25].

As with the cyanobacterium, there are distinct differences in the patterns between the algal species. In Figure 3(a), the brown algae* Ectocarpus* sp. shows multiple small peaks immediately followed by a single large peak at 13 minutes and multiple smaller peaks at 13.8 minutes.* Mougeotia* sp. has a small peak at 12.5 minutes followed by multiple larger peaks at 13.5 minutes, while* Chlorella vulgaris* (*C. vulgaris*) has a single small forward peak just before the 14-minute migration time, and* Ulothrix fimbriata* (*U. fimbriata*) has only a very small peak at 15.5-minute migration time.* Scenedesmus quadricauda* (*S. quadricauda*) has a small peak at 13 minutes and* Oedogonium foveolatum* (*O. foveolatum*) has multiple very small peaks from 13 to 15 minutes. Interestingly, the early migrating peaks are almost all for the forward strand of DNA.

In Figure 3(b) both* Mougeotia* sp. and* C. vulgaris* have a large forward strand peak very close to the 23-minute migration point, as does the brown algae* Ectocarpus* sp., while* U. fimbriata* has two smaller peaks at 21.5 and 24 minutes.* S. quadricauda* has two slightly larger forward peaks at 22 and 23 minutes, and* O. foveolatum* has two small forward peaks at 20 and 21.5 and a larger forward peak at 24 minutes. In the reverse DNA strand,* O. foveolatum*,* U. fimbriata*,* C. vulgaris*, and* Mougeotia* sp. all have a similar peak around 26 minutes, while* S. quadricauda* and* Ectocarpus* sp. have several smaller peaks around 25 minutes.

**Figure 4:**
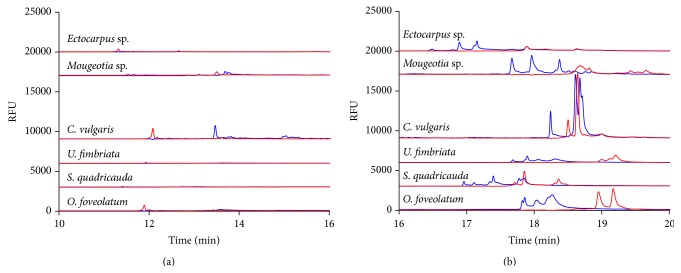
Detection of the (a) early and (b) later migrating peaks formed from the 18S rDNA variable region 9 as detected by CE-SSCP for the green and brown algae. Forward or sense strands are represented in blue while reverse or antisense strands are represented in red.

The forward and reverse strands of the DNA give a pattern that is repeatable but in some cases may not differentiate enough between very similar species. This is the reasoning behind using at least two different variable regions; if one region cannot differentiate decisively between the patterns a second region may be examined. In Figure 4, a different variable region (variable region 9) was explored for these same six algae. In Figure 4(a),* Ectocarpus* sp. has small reverse peaks at 11.5 minutes,* Mougeotia* sp. has a reverse peak at 13.5 minutes, and* C. vulgaris* and* O. foveolatum* have small reverse peaks around 12 minutes.* C. vulgaris* also has a significant forward peak around 13.5 minutes. There are no early migrating peaks for* S. quadricauda* or* U. fimbriata*. In Figure 4(b),* Ectocarpus* sp. has a small reverse peak at 18 minutes, while* Mougeotia* sp. has multiple, larger peaks at 18.5 and 19.5 minutes.* C. vulgaris* has a large reverse peak at 18.5 minutes, while* U. fimbriata* has small multiple peaks at just past 19 minutes.* S. quadricauda* has three small reverse peaks just before 18 minutes, and* O. foveolatum* has two larger peaks at 19 minutes. The forward strands of DNA also have quite different patterns.* Ectocarpus* sp. has three small forward peaks at 17 minutes, while* Mougeotia* sp. has three forward peaks at 17.8-, 18-, and 18.3-minute migration time.* C. vulgaris* has a larger peak at 18.5 minutes followed closely by a triple peak pattern (somewhat obscured by the reverse peak overlaid on top of them) at just before the 19 minutes.* U. fimbriata* has small multiple peaks just after the 18-minute mark and* S. quadricauda* has four small forward peaks through 17 to 18, while* O. foveolatum* has a larger triple peak around 18 minutes.

The advantages of CE-SSCP are the rapid analysis and clear differences in peak patterns. These changes in pattern could be used to detect changes in an algal population over time, effectively giving a “snapshot” of changes in either a closed or open system. The pattern is highly repeatable; in other words, the nucleotide sequence determines the shape and therefore the mobility of the conformers and this shape does in fact give a specific pattern that can be considered a “fingerprint.” Using the two different variable regions, we were able to show differences in the peak patterns of both prokaryotes and eukaryotes. Even very similar algal species had distinct, repeatable patterns. While the pattern or fingerprint is unique to the algal DNA sequence, there is not necessarily just one peak for each sequence. There are several sequences that give multiple peaks patterns. These peaks could indicate that the “pure” cultures are not genetically pure or more likely represent different conformers from the same sequence.

## 4. Conclusions

CE-SSCP is a relatively fast, easy, and inexpensive method for determining changes in a small section of DNA such as a variable region of the 16S or 18S rDNA gene of the small subunit of the ribosome. In this work, CE-SSCP demonstrated the ability to differentiate between two different blue-green algae, five different green algae, and one brown algal species. The “fingerprint” patterns obtained through CE-SSCP of two variable regions from the 16S rDNA gene and two variable regions from the 18S rDNA gene were highly repeatable and unique to each species. This technique shows significant potential for being differentiating algae and could be used to monitor an algal system over time.

## Figures and Tables

**Table 1:** Pure cultures for fingerprint database.

Genus and species	Class, type	Source/catalog number
*Chlorella vulgaris *	Chlorophyceae, green algae	UTEX 395
*Ulothrix fimbriata *	Chlorophyceae, green algae	UTEX LB 638
*Scenedesmus quadricauda *	Chlorophyceae, green algae	UTEX B 614
*Oedogonium foveolatum *	Chlorophyceae, green algae	UTEX LB 933
*Mougeotia *sp.	Chlorophyceae, green algae	UTEX 758
*Ectocarpus *sp.	Phaeophyceae, brown algae	UTEX LB 1433
*Anabaena inaequalis *	Cyanophyceae, cyanobacterium	UTEX B 381
*Nostoc muscorum *	Cyanophyceae, cyanobacterium	UTEX B 486

**Table 2:** Primer information.

Primer name	Gene amplified	Variable region	Primer sequence	Fragment size^*∗*^
V2F [14]	16S	V2	GGCGAACGGGTGAGTAA	239 bp
V2R [14]	16S	V2	ACTGCTGCCTCCCGTAG	239 bp
V3F [15]	16S	V3	CCAGACTCCTACGGGAGGCAG	184 bp
V3R [15]	16S	V3	CGTATTACCGCGGCTGCTG	184 bp
V7F	18S	V7	AACTTAAAGGAATTGACGGAA	156 bp
V7R	18S	V7	GCATCACAGACCTGTTATTGCCCC	156 bp
V9F	18S	V9	GTACACACCGCCCGTCGCACC	310 bp
V9R	18S	V9	TTCCGCAGGTTCACCTACGGA	310 bp

^*∗*^Fragment size estimated from NCBI (http://www.ncbi.nlm.nih.gov).
